# Feature Extraction and Classification on Esophageal X-Ray Images of Xinjiang Kazak Nationality

**DOI:** 10.1155/2017/4620732

**Published:** 2017-04-04

**Authors:** Fang Yang, Murat Hamit, Chuan B. Yan, Juan Yao, Abdugheni Kutluk, Xi M. Kong, Sui X. Zhang

**Affiliations:** ^1^Department of Medical Engineering, The Affiliated Tumor Hospital, Xinjiang Medical University, Urumqi 830011, China; ^2^College of Medical Engineering Technology, Xinjiang Medical University, Urumqi 830011, China; ^3^Department of Radiology, The First Affiliated Hospital, Xinjiang Medical University, Urumqi 830054, China

## Abstract

Esophageal cancer is one of the fastest rising types of cancers in China. The Kazak nationality is the highest-risk group in Xinjiang. In this work, an effective computer-aided diagnostic system is developed to assist physicians in interpreting digital X-ray image features and improving the quality of diagnosis. The modules of the proposed system include image preprocessing, feature extraction, feature selection, image classification, and performance evaluation. 300 original esophageal X-ray images were resized to a region of interest and then enhanced by the median filter and histogram equalization method. 37 features from textural, frequency, and complexity domains were extracted. Both sequential forward selection and principal component analysis methods were employed to select the discriminative features for classification. Then, support vector machine and *K*-nearest neighbors were applied to classify the esophageal cancer images with respect to their specific types. The classification performance was evaluated in terms of the area under the receiver operating characteristic curve, accuracy, precision, and recall, respectively. Experimental results show that the classification performance of the proposed system outperforms the conventional visual inspection approaches in terms of diagnostic quality and processing time. Therefore, the proposed computer-aided diagnostic system is promising for the diagnostics of esophageal cancer.

## 1. Introduction

Esophageal cancer is the eighth most common malignancy worldwide, with more than 480,000 new patients diagnosed annually. According to the Surveillance, Epidemiology, and End Result (SEER) statistics, the 5-year survival rate for esophageal cancer based on stage at diagnosis (2001–2007) is 17% overall: 37% for local disease; 18% for regional disease; and 3% for distant disease [1]. The World Health Report 2004 ranked esophageal cancer as the highest cause of cancer mortality in China. Among the 446,000 causes of death caused by esophageal cancer worldwide, more than half occurred in China, that is, 288 thousand (WHO, 2004) [2–4]. Xinjiang Uygur Autonomous Region is a high incidence area of esophageal cancer. The mortality rate of esophageal cancer for Kazak nationality is 155.9 out of 100,000, which is significantly higher than the average mortality of 15.23 out of 100,000 in China [5]. Over 80% of esophageal cancer occurs in developing countries, where nearly all cases are esophageal squamous cell carcinoma (ESCC). A number of risk factors for ESCC, including tobacco smoking, alcohol drinking, dietary and micronutrient deficiencies, high temperature of beverage and food consumption, and other miscellaneous factors (such as fast eating habits and polycyclic aromatic hydrocarbon exposure), have been identified over the past few decades [6]. The incipient symptoms of esophageal cancer are too inconspicuous to be found. Most patients are diagnosed late in the course of the disease, and at this stage, it carries a bad prognosis. X-ray barium technology, as a crucial tool for the detection of esophageal cancer, offers the specialist physician high-quality visual information to identify the disease types [7]. Classically, the X-ray images are examined manually by physicians, and it is inevitability difficult to avoid inconsistent interpretations by interobservers. In some cases, even for experienced radiologists, they may misinterpret images of the esophageal cancer regions and miss smaller lesions. Therefore, the primary preventive strategies and control activities on esophageal cancer should be enhanced in the future, which are potentially effective to reduce the mortality of esophageal cancer and also essential to save lives and resources. In this paper, a computer-aided diagnostic system is developed to assist physicians in classifying the esophageal cancer with specific disease types.

With the rapid development in computer technology, CAD is currently widely used in the diagnosis or quantification of various diseases [8–10]. Many studies have shown that CAD has the potential to increase the sensitivity and the specificity of diagnostic imaging [11, 12]. The merit of CAD of image features lies in the objectivity and reproducibility of the measures of specific features. The conventional paradigm envisions that the CAD output will be used by the physician as a second opinion with the final diagnosis to be made by the physician [13]. Qi et al. developed a computer-aided diagnosis system to assist the detection of dysplasia in Barrett's esophagus. Experimental results showed that the proposed CAD algorithms had the potential to quantify and standardize the diagnosis of dysplasia and allowed high throughput image evaluation for endoscopic optical coherence tomography screening applications [14, 15]. Sommen et al. presented a novel algorithm for automatic detection of early cancerous tissue in HD endoscopic images. Experimental results showed that of 38 lesions indicated independently by the gastroenterologist, the system detected 36 of those lesions with a recall of 0.95 and a precision of 0.75 [16]. Schoon et al. proposed a CAD system to find the early stages of esophageal cancer. The results showed that the proposed system achieved a classification accuracy of 94.2% on normal and tumorous tissue and reached an area under the curve of 0.986 [17]. Esophageal cancer CAD literature published to data mostly focuses on endoscopic images. In addition to our previous study, no other papers have been found in the field of esophageal X-ray images to our best of knowledge.

The algorithms in the published CAD literature included image preprocessing, feature extraction, and pattern classification. Histogram equalization algorithm is one of the most widely used techniques for enhancing image contrast for its simplicity and effectiveness. Shang et al. proposed a Range Limited Peak-Separate Fuzzy Histogram Equalization (RLPSFHE) for enhancing image contrast for its simplicity and effectiveness. The experimental results show that the RLPSFHE can achieve a better trade-off between mean brightness preservation and contrast enhancement [18]. Zohair et al. introduced an ameliorated version of the contrast-limited adaptive histogram equalization (CLAHE) to provide a good brightness with decent contrast for CT images, which provided acceptable results with no visible artifacts and outperformed the comparable techniques [19]. The purpose of feature extraction is to extract the relevant features from the region of interest as the input vectors of the classifiers. Gu et al. proposed a new feature extraction method called adaptive slow feature discriminant analysis (ASFDA) in order to address the weaknesses of the traditional SFDA. Experimental results proved the superiority of ASFDA among some state-of-the-art methods [20]. Mueen et al. extracted three levels of features global, local, and pixel and combined them together in one big feature vector that achieved a recognition rate of 89% [21].

The classification based on multiple image features has the advantage of increasing accuracy via increasing the amount of information used. However, making use of too many image features derived from a limited training data set increases the risk of overfitting, which will decrease the robustness of the system when classifying data outside of the training set [22]. Therefore, it is necessary to select a limited number of image features to balance accurate and robust classification. Gladis et al. applied principal component analysis (PCA) with support vector machine (SVM) to classify the brain MR images by type. The recognition performance of the proposed technique was compared with three other method systems. Experimental results showed the PCA with SVM outperformed the three other methods in terms of classification accuracy [45]. Li et al. utilized the sequential forward selection algorithm (SFS) to figure out the nonunique probe selection problem. The experimental results demonstrate the proposed method outperformed the other greedy algorithms [23]. Techniques such as artificial intelligence and data mining techniques were widely used in the field of medical imaging classification [24]. SVM is a state-of-the-art pattern recognition technique grown up from a statistical learning theory. Papadopoulos et al. implemented artificial neural network (ANN) and a SVM to characterize the microcalcification clusters in digitized mammograms. The results indicated that the classification performance of SVM is superior to the ANN [25]. Zhu et al. employed the SVM to make a distinction within a class of Src kinase inhibitors. The sequential forward selection and sequential backward selection methods were used to remove redundant variables. The results showed that the proposed method could be employed to structure activity relationship modeling with much improved quality and predictability [37]. Katsuyoshi and Alberto detailed the *K*-nearest neighbor method for the application in breast cancer diagnosis. Experimental results showed that the classification accuracy changes with the number of neighbors and also with the percentage of data used for classification [26]. Chen et al. applied the KNN to classify the lung sounds. Experimental results indicated that the error in respiratory cycles between measured and actual values was only 6.8%, illustrating the potential of the detector for home care application [27]. Sharma and Khanna proposed a CAD system to detect abnormalities or suspicious areas in breast X-ray images and classify them as malignant and nonmaligant. Experiments were performed with three texture feature extraction techniques, including Zernike moments, gray-level co-occurrence matrix, and discrete cosine transform. Experimental results showed that SVM with Zernike moments attains the optimum performance [28]. Though the literature published has shown the superiority on the recognition performance of the SVM and KNN, the impact of various feature selection algorithms on classification performance has not been fully explored.

This paper presents a computer-aided diagnostic system to classify the medical X-ray images of Xinjiang Kazak nationality esophageal by type. The proposed system consists of (I) image preprocessing, (II) feature extraction, (III) feature selection, and (IV) classification and performance evaluation. Firstly, the original images are resized to a region of interest and then enhanced by the median filter and histogram equalization method. During the feature extraction and selection step, the feature vectors of the classifiers are selected by PCA and SFS among 37 features in the textural, frequency, and complexity domains. The employed classifiers, that is, SVM and KNN, are validated using a 10-fold cross-validation technique that yields an average estimation of classifier performance with 95% confidence intervals. The performances of both classifiers are investigated with and without prior PCA and SFS input feature vector selection. AUC values of the receiver operating characteristic (ROC) curves, accuracy, precision, and recall, are used to evaluate the classification performance.

## 2. Methods and Techniques

**Figure 1:**
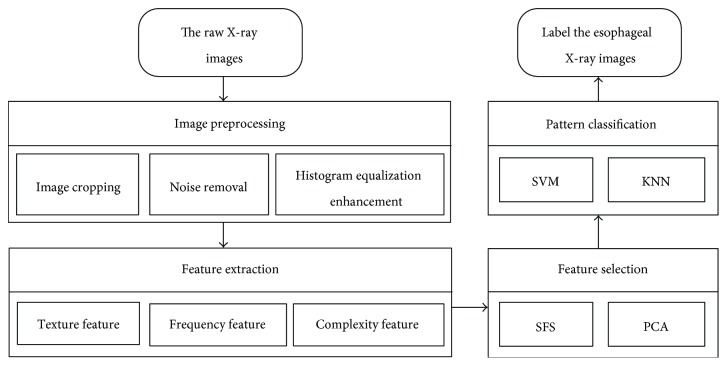
Flow chart of the system design.

The proposed methodology is applied to 300 raw esophageal X-ray images, of which 100 were classified by a pathologist as normal images and 200 as abnormal images. The abnormal cases were further divided in two categories: 100 fungating type and 100 ulcerative type. These images, which included 221 males (mean age: 65) and 79 females (mean age: 68) with an age range of 45–80 years, were collected from The First Affiliated Hospital, Xinjiang Medical University of China. The proposed algorithms were implemented in the Matlab 2013 platform. The flow chart of the system design is depicted in Figure 1.

### 2.1. Image Preprocessing

**Figure 2:**
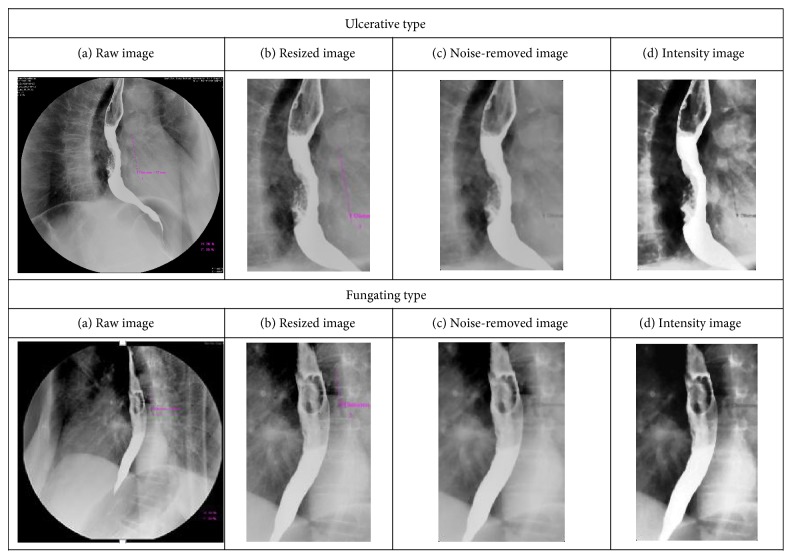
Preprocessing results of the abnormal esophageal X-ray images.

Customarily, preprocessing is a necessity whenever the data to be mined is noisy, inconsistent, or incomplete. Preprocessing significantly improves the effectiveness of data mining techniques [29]. The typical size of the raw images is 1012 × 974, and almost 50% of the whole image comprised the background with a lot of noise. Moreover, these images are scanned at different illumination conditions, so some images appeared too bright and some are too dark. To circumvent the above-mentioned issue, the first step toward noise removal is pruning the original images with a cropping operation. The images are resized to a region of interest of 140 × 240 pixels, which can guarantee that all the regions of interest contain the lesion areas meanwhile avoid the useless information. In addition, the median filter is applied to the cropped images in order to further eliminate the image noise. The second step is image enhancement, in particular, the histogram equalization method, which can increase the contrast range in an image by increasing the dynamic range of gray levels, which is utilized to enhance the image for diminishing the effects of over-brightness and over-darkness in images. The preprocessed images are again inspected by a pathologist to ensure that their quality was sufficient for diagnosis.  Figure 2 presents the preprocessing results of the abnormal esophageal X-ray images, fungating and ulcerative esophageal X-ray images.

### 2.2. Feature Extraction

The purpose of feature extraction in this project is to convert a two-dimensional image into a feature vector, which can be further utilized as the input for the mining phase of the classifier. The extracted features should provide the characteristics of the input type to the classifier by considering the description of the relevant properties of the image into feature vectors. Accordingly, three kinds of features are extracted to describe the structure information of texture, frequency, and complexity.

#### 2.2.1. Texture Features

Texture contains important information regarding underlying structural arrangement of the surface of an image. Gray-level co-occurrence matrix (GLCM), which describes patterns of gray-level repetition, is a well-known texture extraction method originally introduced by Haralick et al. [30]. The co-occurrence matrix is constructed by getting information about the orientation and distance between the pixels. Assuming that *f*(*x*, *y*) is a two-dimensional image with the size of *M* × *N*, the definition of the co-occurrence matrix is as follows:

(1) $$\begin{array}{c} P\left(i,j\;\left|\;d,\theta \right.\right)=\#\left\{\left[\left(x1,y1\right),\left(x2,y2\right)\right]\;\in \;M\times N\;\left|\;d,\theta ,f\left(x1,y1\right)=i,f\left(x2,y2\right)=j\right.\right\}, \end{array}$$

where #{} denotes the number of the elements of the set. *d* and *θ* are the distance and angle between (*x*1, *y*1) and (*x*2, *y*2), respectively.

Many texture features can be directly computed from the gray-level co-occurrence matrix. Pourghassem et al. extracted contrast, correlation, energy, and homogeneity from GLCM [31].

(2) $$\begin{array}{c} \text{Contrast}=\overset{L-1}{\underset{i=1}{\sum }}\overset{L-1}{\underset{j=1}{\sum }}{\left(i\;-\;j\right)}^{2}P\left(i,j,d,\theta \right),\\ \text{Correlation}=\frac{\sum_{i=1}^{L-1}\sum_{j=1}^{L-1}i\cdot j\cdot P\left(i,j,d,\theta \right)-\mu_{x}\mu_{y}}{\sigma_{x}\sigma_{y}},\\ \text{Energy}=\overset{L-1}{\underset{i=1}{\sum }}\overset{L-1}{\underset{j=1}{\sum }}{\left[P\left(i,j,d,\theta \right)\right]}^{2},\\ \text{Homogeneity}=\overset{L-1}{\underset{i=1}{\sum }}\overset{L-1}{\underset{j=1}{\sum }}\frac{p\left(i,j,d,\theta \right)}{1+\left|i-j\right|}, \end{array}$$

where (*μ*_*x*_, *σ*_*x*_) and (*μ*_*y*_, *σ*_*y*_) are mean and standard deviation of pixel value in the row and column directions of the GLCM, respectively. For this task, we calculate a gray-level co-occurrence matrix for four different directions *θ*  ∈  {0°, 90°, 45°, and 135°} and the distance *d* = 1. As a result, texture feature vector includes 16 elements.

#### 2.2.2. Frequency Features

**Figure 3:**
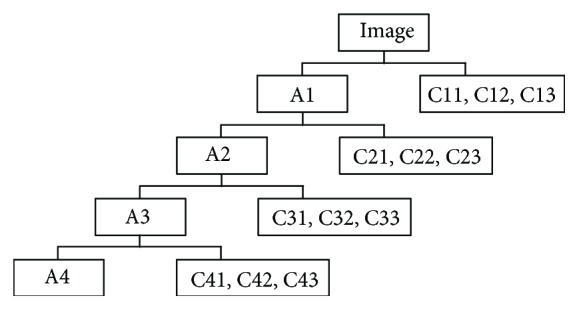
Four-level DWT decomposition process.

The discrete wavelet decomposition (DWT) has been widely used as a fast algorithm to obtain the wavelet transform of X-ray medical images [32, 33]. The DWT analyzes the images by decomposing it into coarse approximation and detailed information representing the low- and high-frequency contents of images, respectively. The approximation can be further calculated to produce the approximation and detailed information at the next level of the decomposition and so on till the required level is reached.  Figure 3 depicts the wavelet decomposition process of this work. Specifically, A1–A4, representing the wavelet approximations of four levels, are low-frequency part of the images. C11–C13, C21–C23, and C31–C33, denoting the details of horizontal, vertical, and diagonal directions of four levels, are high-frequency part of the images. Empirically, C11–C13 can be discarded, since they contain little useful information and a lot of noise. And the approximation coefficient A4 at fourth level is used to represent the low frequency of the image. The mean and variance values are further calculated from each coefficient after the DWT is performed on the X-ray images. Therefore, 20 features are extracted from an input image.

#### 2.2.3. Kolmogorov Complexity Features

An image can be converted into a one-dimensional binary sequence via scanning it either horizontally or vertically. The complex value of each row vector can be obtained by evaluating the complexity of each vector in the horizontal direction. The complexity of the complex vector, which is comprised of the complexity of each row, can be calculated as the complexity feature of the image. Kolmogorov [34] proposes to measure the conditional complexity of a finite object *x*, given a finite object *y* by the length of the shortest sequence *p*, that consists of 0 s and 1 s and thus makes it possible to reconstruct *x* given *y*. Mathematically, this is explained as follows:

(3) $$\begin{array}{c} K_{B}\left(x\;\left|\;y\right.\right)=min\left\{l\left(p\right)\;\left|\;B\left(p,y\right)=x\right.\right\}, \end{array}$$

where *l*(*p*) is the length of the sequence *p*  and  *B*(*p*, *y*) is the decoding function, for which there is an algorithm computing its values.

Kolmogorov only gave a general definition of the Kolmogorov complexity. Kasper and Schuster [35] proposed an explicit algorithm to compute the KC measure, which includes two operations, copying and inserting. After the explicit algorithm is applied to the images, one feature is obtained.

### 2.3. Feature Selection

Feature selection is an optimization technique that, given a set of features, attempts to select a subset of size that leads to the maximization of some criterion function [36]. In this paper, we employ both sequential forward selection (SFS) and principal component analysis (PCA) methods to select the discriminative features among the feature vector.

#### 2.3.1. Sequential Forward Selection

Informally, SFS algorithm can be described as follows [37]: SFS begins with an empty feature set, and all the observation features were marked as nonselected features. At each iteration, one feature from among the nonselected features is added to the feature set, which minimizes the mean square error (MSE). The iterative process could be stopped until the best merit MSE is obtained. MSE can be defined as follows:

(4) $$\begin{array}{c} \text{MSE}=\frac{1}{N}\overset{N}{\underset{i=1}{\sum }}\left(x_{i}-\bar{x}\right), \end{array}$$

where *X* denotes the random variables. *N* is defined as the number of samples taken.

#### 2.3.2. Principal Component Analysis

Principal component analysis, which is also known as Karhunen-Loeve (KL) transform, is a projection-based technique that facilitates a reduction in data dimension through the construction of orthogonal principal components that are weighted, linear combinations of the original variables [38–40]. Assuming that a linear transformation mapping the original *N*-dimensional feature space into an *M*-dimensional space, where *M* < *N*, the PCA transform can be denoted as follows:

(5) $$\begin{array}{c} F_{D}=F_{V}X_{a}, \end{array}$$

where *F*_*V*_ is the so-called eigenvector, whose length depends on the components that we want for expressing the observation feature space. The resultant feature space is the projection of the original data set over the eigenvectors of the covariance matrix. In this study, we applied the PCA for investigating if the reduced set of features can retain significant discrimination of the projected data. Firstly, the original matrix was converted into a standardized matrix. That is, the features were normalized to have zero means and unit variances. Secondly, the covariance matrix, which comprises the weights of each feature in the input space, was calculated. In addition, the eigenvalues and the corresponding eigenvectors of the covariance matrix were computed. The eigenvector with highest eigenvalue was the first principle component that contains the most significant information and accounts for the larger amount of variance in the data. The first few principal components are selected to be the inputs of classifiers when their accumulative contributive rate was 0.9.

### 2.4. Classification and Performance Evaluation

In this study, two classifiers, that is, *K*-nearest neighbors (KNN) and support vector machine (SVM) with radial basis function (RBF), were used for classification. SVM seeks the optimal boundary between two classes. The popularity of this method has grown as it provides a powerful machine learning technique to classify data. KNN is known in the machine learning field as a nonparametric method.

#### 2.4.1. Support Vector Machine (SVM)

Support vector machine, a technique derived from statistical learning theory, is the most promising technique for data classification and regression and function estimation [41–44]. The basic idea of applying SVM for solving classification problems can be stated briefly as follows: (a) transform the input space to higher dimension feature space by a nonlinear mapping function and (b) construct the separating hyperplane with the maximum distance from the closest points of the training set [45]. SVM has high classifying accuracy and good capabilities of fault tolerance and generalization. SVM constructs a binary classifier from a set of training samples (*x*_1_,…, *x*_*n*_), which belongs to a class label. SVM selects the hyperplane that causes the largest separation among the decision function values for the borderline examples of the two classes. The hyperplane decision function can be defined as follows:

(6) $$\begin{array}{c} f\left(x\right)=\text{sign}\left[\overset{n}{\underset{i=1}{\sum }}\alpha_{i}y_{i}K\left(x_{i},x\right)-b\right], \end{array}$$

where *K*(*x*_*i*_, *x*) is the kernel function. *b* is the classification threshold. *α*_*i*_ is lagrangian multiplier, which is calculated by quadratic programming problem.

(7) $$\begin{array}{c} \underset{\alpha }{max}W\left(\alpha \right)=\overset{l}{\underset{i=1}{\sum }}\alpha_{i}-\frac{1}{2}\overset{l}{\underset{i=1}{\sum }}\overset{l}{\underset{j=1}{\sum }}\alpha_{i}\alpha_{j}y_{i}y_{j}K\left(x_{i},x_{j}\right) \end{array}$$

subject to ∑_*i*=1_^*l*^*α*_*i*_*y*_*i*_ = 0, 0 ≤ *α*_*i*_ ≤ *C* (*i* = 1,…, *l*), 0 ≤ *α*_*j*_ ≤ *C* (*j* = 1,…, *l*).

There are three parameters in SVM model that we should choose. They make great impact on a model's generalization ability. It is well known that SVM generalization performance depends on a good setting of hyperparameters *C*, the kernel function, and kernel parameter. For multiclassification problems, there are two general approaches, one-against-one and one-against-all. In the former approach, classifier is calculated from each pair of classes. All classifiers are combined to conclude the final classification by using majority voting scheme. In the latter one, the classifier is calculated from each class versus all classes and then the first object that is classified as a single class is the type of the unlabeled data.

#### 2.4.2. *K*-Nearest Neighbors (KNN)

The *K*-nearest neighbor classifier is firstly proposed by Cover and Hart in 1968 [46]. It is a nonparametric learning algorithm that is used for classification and regression [47]. KNN is a very simple but efficient algorithm because it is a typical type of instance-based or memory-based learning scheme. The implementation process of the *K*-nearest neighbor algorithm is as follows [48]:

- In the first step, the number of nearest points of test data *x* against training data *K* is determined. Euclidean distance is the most commonly used to measure the distance between two instances according to the type of attribute [49]. Assuming there are two points in *K*-dimensional space, *x* = [*x*_1_, *x*_2_,…, *x*_*k*_] and *y* = [*y*_1_, *y*_2_,…, *y*_*k*_], the Euclidean distance between the two can be denoted by

(8) $$\begin{array}{c} d\left(x,y\right)=\sqrt{\overset{k}{\underset{i=1}{\sum }}{\left(y_{i}\;-\;x_{i}\right)}^{2}}. \end{array}$$

(II) We can judge that the test data *x* is a certain category when it has more representatives than a certain category of data.

Generally, larger values of *k* reduce the effect of noise on the classification, but make boundaries between classes less distinct. A good *k* can be selected by cross-validation, running the nearest neighbor classifier on the learning set only. Due to its implementation simplicity and classification effectiveness, KNN has been widely used in pattern recognition. It is also used as a different feature selection algorithm [50, 51] and is integrated into the feature selection framework to evaluate the quality of a candidate feature subset [52–54].

#### 2.4.3. Performance Evaluation

The classifiers are validated using a 10-fold cross-validation technique that yields an average estimation of classifier performance with 95% confidence intervals. In the cross-validation, 90% of samples were used for training and 10% were used for the validation replications. The performances of the classifiers are evaluated in terms of the area under the receiver operating characteristic (ROC) curve (AUC), accuracy, precision, and recall. The ROC analysis is a commonly used approach for classification performance evaluation [55]. The AUC value is the average true positive rates over all possible false positive rates. The accuracy, precision, and recall [56] are given as follows:

(9) $$\begin{array}{c} Accuracy=\frac{Number\;of\;correctly\;classified\;images}{Total\;Number\;of\;images}\times 100\%,\\ Precision=\frac{Number\;of\;correctly\;classified\;images\;per\;class}{Total\;number\;of\;classified\;images\;per\text{ class}}\times 100\%,\\ Recall=\frac{Number\;of\;correctly\;classified\;images}{Total\;number\;of\;expected\;images\;in\;the\;corresponding\;class}\times 100\%. \end{array}$$

## 3. Results and Discussion

The above-described methodology has been evaluated on a set of esophageal X-ray images collected from The First Affiliated Hospital of Xinjiang Medical University. During the classification stage, performance comparison is divided into three categories: (1) all 37 features; (2) features selected by SFS; and (3) features selected by PCA. The classification was conducted on a two-stage process. In the first-stage classification process, the X-ray images are classified as normal and abnormal. Then the second-stage classification process continues the abnormal images that are classified as fungating and ulcerative type images. And the classifiers were validated by a 10-fold cross-validation technique. The classification performance was measured by the AUC values of the ROC curves, accuracy, precision, and recall.

Feature selection is carried out using SFS and PCA methods to remove the redundancy due to highly correlated features. During the first-stage and second-stage classification processes, the SFS selected 17 appropriate features out of 37 features, respectively. It means a reduction of computing time and data storage space. The selected features are from the textural, frequency, and complexity domains and all useful for the classification. The results of feature selection of SFS for the two-stage classification process are detailed in Tables 1 and 2. Among the appropriate 17 features selected by the SFS, the higher proportion is *θ* = 45°, 90°. This result shows that texture of esophageal focus may occur in the particular angle and distance. Each principal component is orthogonal and represents a linear combination of the original variables. The first few principal components typically account for most of the variance in the original data. In this analysis, the first six principal components together explained 90.7% and 92.26% of the variance for the first-stage and second-stage classification processes, respectively. The eigenvalue and the cumulative variance of the first six principal components for the two-stage classification are tabulated in Table 3.

**Figure 4:**
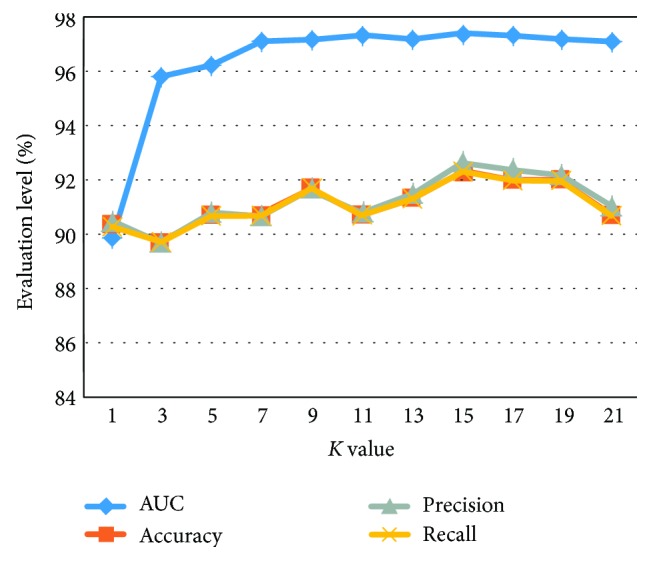
KNN classification results for various choices of *K* (%).

Figure 4 reports the KNN classification results for values of *K* ranging from one to twenty-one using 10-fold cross-validation. It can be seen from Figure 4 that KNN classifier achieved the best classification when *K* = 15. It is observed that the KNN classifier has an AUC value of 97.4%, accuracy of 92.33%, precision of 92.7%, and recall of 92.3%.

**Figure 5:**
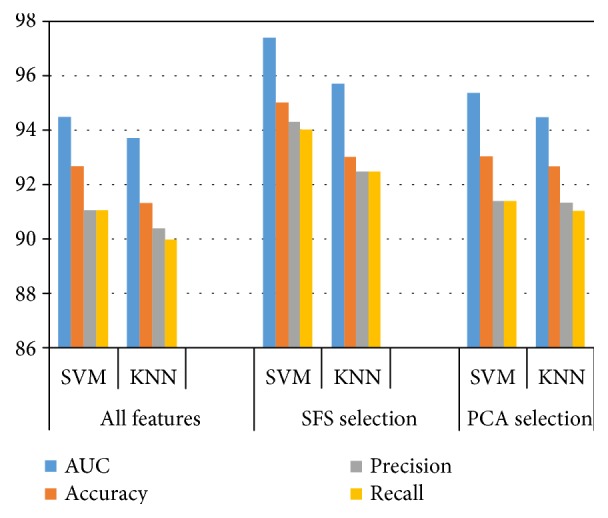
Classification performance of the first classification stage (%).

**Figure 6:**
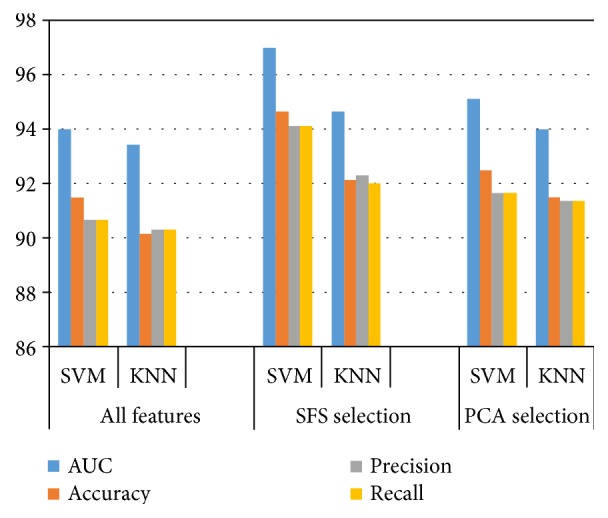
Classification performance of the second classification stage (%).

The radial basis function (RBF) kernel is chosen for SVM classifier. For the training of KNN classifier, the number of the nearest neighbor *K* = 15 and Euclidean distance metric was employed. Based on the result shown in Table 4, Figure 5, and Figure 6, the following conclusions can be drawn:

- The step of feature selection not only reduces the dimension of the input vector, but also improves classification performance. This may be due to the elimination of the correlated features from the 37-D feature vector.
- The SFS outperforms the PCA. In the first-stage classification, for all 37 features used as input vectors, it yields the best AUC value of 94.5%, accuracy of 92.67%, precision of 91%, and recall of 91%. With input features selected by SFS and PCA, the corresponding AUC value, accuracy, precision, and recall are 97.4% and 95.33%, 95% and 93%, 94.33% and 91.4%, and 94% and 91.4%, respectively. In the second-stage classification, it produces the best AUC value of 94%, accuracy of 91.5%, precision of 90.67%, and recall of 90.67% for all the 37 features. With the input vectors selected by SFS and PCA, the corresponding AUC value, accuracy, precision, and recall are 97% and 95.14%, 94.67% and 92.5%, 94.14% and 91.67%, and 94.14% and 91.67%, respectively.
- Under either feature selection criterion (no selection, SFS selection, and PCA selection), the performance of SVM is better than the KNN. The highest classification performance was achieved when the SVM classifier and SFS selection are employed.

In our previous studies, several methods related to computer-aided diagnosis system of esophageal cancer have been developed. The classification performances are tabulated in Table 5. It is observed that single feature reached lower classification accuracy. The classification performance improved in the case of using the comprehensive feature without dimensional reduction algorithm. When the feature extraction methods were utilized, the accuracy obtained the further improvement.

Although the previous works have made some achievements, the classification performance still needs to be improved in order to meet the requirements of esophageal cancer diagnosis. The present study introduced the KC feature extraction and SFS and SVM algorithms, and the high classification performance was achieved by combining with the previous method.

The processing time of the proposed method takes around 14.32 s (11.02 s for image preprocessing, 2.16 s for feature extraction, and 1.14 s for classification) while the manual recognition takes about 37 s. The accuracy of detecting the esophageal cancer via both specialist physicians and the proposed method is 92% and 95%, respectively. And the accuracy of classifying the abnormal images into fungating and ulcerative types reaches up to 90% and 94.67%, respectively. The classification performance of the proposed method outperforms the conventional visual inspection approach by improving the diagnostic quality and processing time.

## 4. Conclusions

Esophageal cancer has a high mortality in Xinjiang Kazak nationality. X-ray barium technology is more commonly used in the diagnosis of this disease. However, the differences of experience, knowledge, and skills among individual physicians may affect the diagnosis results. This paper presents a computer-aided diagnosis system with image processing and pattern recognition in diagnosing esophageal cancer of Xinjiang Kazak nationality by using X-ray images. The original images, including normal esophageal images, fungating and ulcerative type images, were first resized to a region of interest and then enhanced by the median filter and histogram equalization method. Then, 37 features were obtained from images using three different techniques, which include textural, frequency, and complexity domains. SFS and PCA methods were applied to select the input features for classification. Furthermore, the esophageal cancer images were classified via SVM and KNN classifiers by type. And the classifiers were validated by a 10-fold cross-validation strategy. The classification performance was evaluated in terms of the AUC values, accuracy, precision, and recall, respectively.

A two-stage classification process was carried out for classifying the esophageal cancer by type. In the first-stage classification process, the X-ray images are classified as normal and abnormal. For all 37 features used as input vectors, it yielded the best AUC value of 94.5%, accuracy of 92.67%, precision of 91%, and recall of 91%. With input features selected by SFS and PCA, the corresponding AUC value, accuracy, precision, and recall were increased by 2.9% and 0.83%, 2.33% and 0.33%, 3.33% and 0.4%, and 3% and 0.4%, respectively. Then the second-stage classification process continues the abnormal images that are classified as fungating and ulcerative type images. It produced the best AUC value of 94%, accuracy of 91.5%, precision of 90.67%, and recall of 90.67% for all the 37 features. With the input vectors selected by SFS and PCA, the corresponding AUC value, accuracy, precision, and recall were increased by 3% and 1.14%, 3.17% and 1%, 3.47% and 1%, and 3.47% and 1%, respectively. Experimental results show that the highest classification performance is achieved when the SVM classifier and SFS selection were employed. The accuracy of detecting the esophageal cancer and classifying it by type via specialist physician and the proposed method is 92% and 95% and 90% and 94.67%, respectively. The classification performance of the proposed system outperformed the conventional visual inspection approach by improving the diagnostic quality and processing time.

The proposed method may be limited in the following aspects. First, the regions of interest of the images were selected manually, which result to be time-consuming during the image processing stage. This is because the lesion areas vary greatly from different images, and it is hard to find a unified segmentation method at present. The second important limitation of the study is the lack of comparison with the early esophageal cancer because of the small number of images in early stage. Based on the limitations of the current study, the future perspectives of our work aiming for diagnostic quality improvements may lie in studying more advanced feature extraction model and the segmentation method for esophageal X-ray images. An interesting improvement could be to extend it into the comparison research between the normal esophageal and the early esophageal cancer.

## Figures and Tables

**Table 1:** Details of feature selection by SFS for the first-stage classification process.

Features		Feature number			
Texture features (*θ*, *d*)	(0°, 1)	*1*	2	3	4
	(45°, 1)	5	*6*	*7*	*8*
	(90°, 1)	*9*	*10*	*11*	12
	(135°, 1)	13	14	15	16

Frequency features		17	18	19	20
		21	22	23	*24*
		*25*	*26*	*27*	*28*
		*29*	*30*	31	32
		33	*34*	*35*	36

KC features		*37*			

The numbers in italics are the features selected by SFS.

**Table 2:** Details of feature selection by SFS for the second-stage classification process.

Features		Feature number			
Texture features (*θ*, *d*)	(0°, 1)	*1*	2	3	4
	(45°, 1)	5	*6*	7	*8*
	(90°, 1)	9	*10*	*11*	12
	(135°, 1)	*13*	14	15	16

Frequency features		17	18	19	20
		*21*	22	*23*	*24*
		25	*26*	*27*	*28*
		*29*	*30*	31	*32*
		33	34	*35*	36

KC features		*37*			

The numbers in italics are the features selected by SFS.

**Table 3:** Details of feature selection by PCA for the two-stage classification process.

PC	Eigenvalue		Cumulative variance (%)	
	First stage	Second stage	First stage	Second stage
PC1	11.07	15.1	35	45.8
PC2	6.84	6.2	53.4	62.56
PC3	5.57	4.44	68.4	74.57
PC4	3.4	3.24	77.6	83.32
PC5	2.94	1.84	85.6	88.3
PC6	1.91	1.47	90.7	92.26

**Table 4:** Classification performance of SVM and KNN classifiers (%).

Parameters		All features		SFS selection		PCA selection	
		SVM	KNN	SVM	KNN	SVM	KNN
AUC	First stage	94.5	93.7	97.4	95.7	95.33	94.5
	Second stage	94	93.4	97	94.67	95.14	94
Accuracy	First stage	92.67	91.3	95	93	93	92.67
	Second stage	91.5	90.14	94.67	92.14	92.5	91.5
Precision	First stage	91	90.4	94.33	92.5	91.4	91.33
	Second stage	90.67	90.33	94.14	92.33	91.67	91.4
Recall	First stage	91	90	94	92.5	91.4	91
	Second stage	90.67	90.33	94.14	92	91.67	91.4

**Table 5:** Classification performance of previous studies (%).

Methods	Classification accuracy
GH + Bayes [57]	76.6
WT + Bayes [58]	76.5
GH + GLCM + Bayes [59]	86.7
GLCM + GGCM + PCA + KNN [60]	87.5

GH: gray-level histogram; WT: wavelet-based transform; GGCM: gray-gradient co-occurrence matrix.
